# The peopling of Lakshadweep Archipelago

**DOI:** 10.1038/s41598-019-43384-3

**Published:** 2019-05-06

**Authors:** Mohammed S. Mustak, Niraj Rai, Mohan Rao Naveen, Satya Prakash, S. Justin Carlus, Nagarjuna Pasupuleti, Anshika Srivastava, Prajjval Pratap Singh, Idrees Babu, Pavan Kumar Dubey, Gyaneshwer Chaubey, Kumarasamy Thangaraj

**Affiliations:** 1 Department of Applied Zoology, Mangalore University, Mangalore, 574199 India; 2 CSIR-Centre for Cellular and Molecular Biology, Uppal Road, Hyderabad, 500007 India; 3 Birbal Sahni Institute of Palaeosciences, 53 University Road, Lucknow, 226007 India; 4 Cytogenetics Laboratory, Department of Zoology, Banaras Hindu University, Varanasi, 221005 India; 5 Department of Science and Technology, Lakshadweep Administration, Kavaratti, 682555 India; 6 Prosthodontics Unit, Faculty of Dental Sciences, Institute of Medical Sciences, Varanasi, 221005 India; 7 Estonian Biocentre, Institute of Genomics, University of Tartu, Tartu, 5100 Estonia

**Keywords:** Population genetics, Evolutionary biology

## Abstract

The archipelago of Lakshadweep is considered as a stopover to the maritime route since ancient time. It is not very clear when the human first occupied these islands, however in the long history of the islands, the local legends suggest that Lakshadweep has been ruled by different kingdoms. To have a better understanding of peopling of Lakshadweep, we have analysed 557 individuals from eight major islands for mitochondrial DNA and 166 individuals for Y chromosome markers. We found a strong founder effect for both paternal and maternal lineages. Moreover, we report a close genetic link of Lakshadweep islanders with the Maldives, Sri Lanka and India. Most of the Lakshadweep islands share the haplogroups specific to South Asia and West Eurasia, except Minicoy Island that also shares haplogroups of East Eurasia. The paternal and maternal ancestries of the majority of island populations suggest their arrival from distinct sources. We found that the maternal ancestry was closer to South Indian populations, whereas the paternal ancestry was overwhelmed with the haplogroups, more common in the Maldives and North of India. In conclusion, our first genetic data suggest that the majority of human ancestry in Lakshadweep is largely derived from South Asia with minor influences from East and West Eurasia.

## Introduction

Lakshadweep is an archipelago of about thirty-five islands, scattered over approximately 78,000 square km of the Arabian Sea, 200–440 kms off the south-western coast of India^1,2^. The literal meaning of Lakshadweep is “one hundred thousand islands” in *Sanskrit*. The total population size of the archipelago is approximately 65 thousands, however, it lacks aboriginal population^1,3^. Presently, majority of the populations follow Islam and are ethnically similar to Malayali people of Kerala state^2^. The first human settlement of this archipelago is not very clear, nevertheless it was under the power of various regime. Amini, Kalpeni Andrott, Kavaratti and and Agatti are the oldest inhabited islands. The islands were known to sailors since ancient time^2^. *Jataka* stories of Buddhism has mentioned these islands, which documented the spread of Buddhism to the islands during 6^th^ century BC^1,4^. The local stories suggest the arrival of Islam in 661 AD by Arabians. Later, Cholas ruled the islands in 11^th^, Portuguese in 16^th^, Ali Rajahs in 17^th^, Tipu Sultan in 18^th^ and finally it was under British Raj in 19^th^ Century^1–6^. Arab traveller *Ibn Batuta* mentioned about these islands in many of his stories^2^.

Due to its geographical location, the present day Lakshadweep populations may offer a unique insight into historic migration events. The exposure of these islands to people from pan-world ethnicity might have created a mosaic like pattern in their genomic ancestry, however no genetic study so far has been done on populations living in these islands. Therefore, for the first time, we have done extensive DNA sampling of several major islands of Lakshadweep (Supplementary Fig. 1), and performed a high-resolution analysis of haploid markers. We looked specifically for the composition of various haplogroups (hg) present in these islands and their intra and inter-relations to the surrounding populations.

## Methods

### Sampling

Approximately, 5–10 ml blood samples, were collected from 557 individuals belonging to eight major islands of Lakshadweep (Agatti, Andorth, Bitra, Chetlat, Kadmat, Kalpeni, Kiltan and Minicoy), with informed written consent (Supplementary Fig. 1). With detailed interview procedure about their family pedigree, we have excluded the samples of the people who are related for minimum of three generations. This project was approved and permitted by the Institutional Human Ethical Committee, Mangalore University, Karnataka, India and Institutional Ethical Committee of the CSIR-Centre for Cellular and Molecular Biology, Hyderabad, India. All methods were performed in accordance with the relevant guidelines and regulations.

### Genotyping

We have used Sanger Sequencing method and utilised 23 F and 23 R markers described elsewhere^7^ to sequence the Hypervariable segment I (HVS-I) of mtDNA. We sequenced both of the strands to minimize errors. Variations were recorded against the r-CRS^8^. To assign those samples in haplogroups, we subsequently genotyped coding region diagnostic mutations and used these combined information to assign haplogroups (Table 1, Supplementary Tables 1 and 2). They were further verified by consulting the data published till date in PhyloTree build 16^9^. In case of Y chromosome, we genotyped 166 samples for 44 biallelic markers published recently^10^ to assign the Y chromosomal haplogroups (Supplementary Table 2). For all the Y chromosome markers, we utilised Sanger sequencing (ABI 3700, Applied Biosystems) method and compared it with the reference to note the variations.

**Table 1:** The distribution of various maternal haplogroups into the islands of the Lakshadweep.

Islands	*n*	M	M2a1	M2b	M2b2	M2c	M3	M30	M33a1a	M35	M3c	M4	M5	M5a2a	M64	M66	M6a	M7c	M9a1b1	R	R30	R31	R5	R8b	U1a1	U2	U2c1	U4	U7	U9a1
Agatti	94	0.02	—	0.01	0.35	—	—	0.15	—	—	—	—	0.01	—	0.04	—	—	—	0.01	0.02	0.38	—	—	—	—	—	—	—	—	—
Androth	96	0.08	—	—	0.06	—	—	0.08	0.01	—	—	0.04	—	—	0.02	—	0.03	—	—	0.55	0.03	—	—	—	—	—	—	0.08	—	—
Bitra	13	—	—	—	0.31	—	—	—	—	—	—	—	—	—	—	—	—	—	—	—	0.62	—	—	—	0.08	—	—	—	—	—
Chetlat	43	—	—	—	0.14	—	—	0.02	—	—	—	—	—	—	—	—	—	—	—	—	0.81	—	—	—	—	—	—	—	0.02	—
Kadmat	74	—	0.04	—	0.14	0.04	—	0.03	—	0.01	—	—	0.01	—	—	—	—	—	—	—	0.68	0.03	—	—	0.03	—	—	—	—	—
Kalpeni	94	0.03	—	—	0.10	—	0.12	0.16	—	—	—	—	0.01	—	0.02	—	—	—	—	0.02	0.14	—	—	—	—	—	—	0.39	0.01	—
Kiltan	70	0.01	—	—	0.40	—	—	0.03	—	—	0.01	—	—	—	—	—	0.07	—	—	0.01	0.34	0.06	0.04	0.01	—	—	—	—	—	—
Minicoy	73	0.05	—	—	—	—	—	—	—	—	—	—	—	0.01	0.05	0.15	0.01	0.12	—	0.10	0.01	0.01	0.01			0.01	0.16	0.03		0.25

### Statistical analysis

For statistical analysis, first we plotted the PC1 vs PC2 estimated by using POPSTR (kindly provided by H. Harpending), to infer the inter and intra relationship of populations. We plotted the regional haplogroup affiliation of all the islands over their geographical co-ordinates. The classification of different haplogroups to East Eurasia, West Eurasia and South Asia was done manually considering few major points e.g. their exclusive presence in a region, their high frequency and diversity in a region and based on classifications used in previous publications^11,12^. In cases where the particular geography was not determined with the current resolution of data, we have put them as haplogroup of ‘unknown’ origin. We also took special care for haplogroup R1a-M17 which harbours substantial amount of Indian paternal ancestry^13^. It is a haplogroup of debatable origin^14,15^, we used it as such. The haplotype diversity and AMOVA tests were performed by using Arlequin 3.5 software^16^ Median-joining and reduced median networks were reconstructed with NETWORK program (version 5)^17^. Reduced median and median-joining procedures were applied sequentially for the analysis and they showed similar network structure. For comparison, we have used various datasets published elsewhere^11,18–29^.

## Results and Discussion

The Lakshadweep islands, were well known to the maritime sailors since ancient times, presumably due to a staging point between different continents^1,2^. The extensive maritime history facilitated the access of these islands by various ethnic groups, therefore, it is likely that the genetic landscape of these islands might have shaped uniquely. However, this assumption has not been investigated at the molecular level that simultaneously examines the ancestry composition of different islands and their genetic relationships that might also co-vary with distance from neighbouring regions. To validate these assumptions, we have analysed 557 samples for mitochondrial DNA (mtDNA) and 166 samples for Y chromosome markers and compared them with the neighbouring regions.

**Figure 1:**
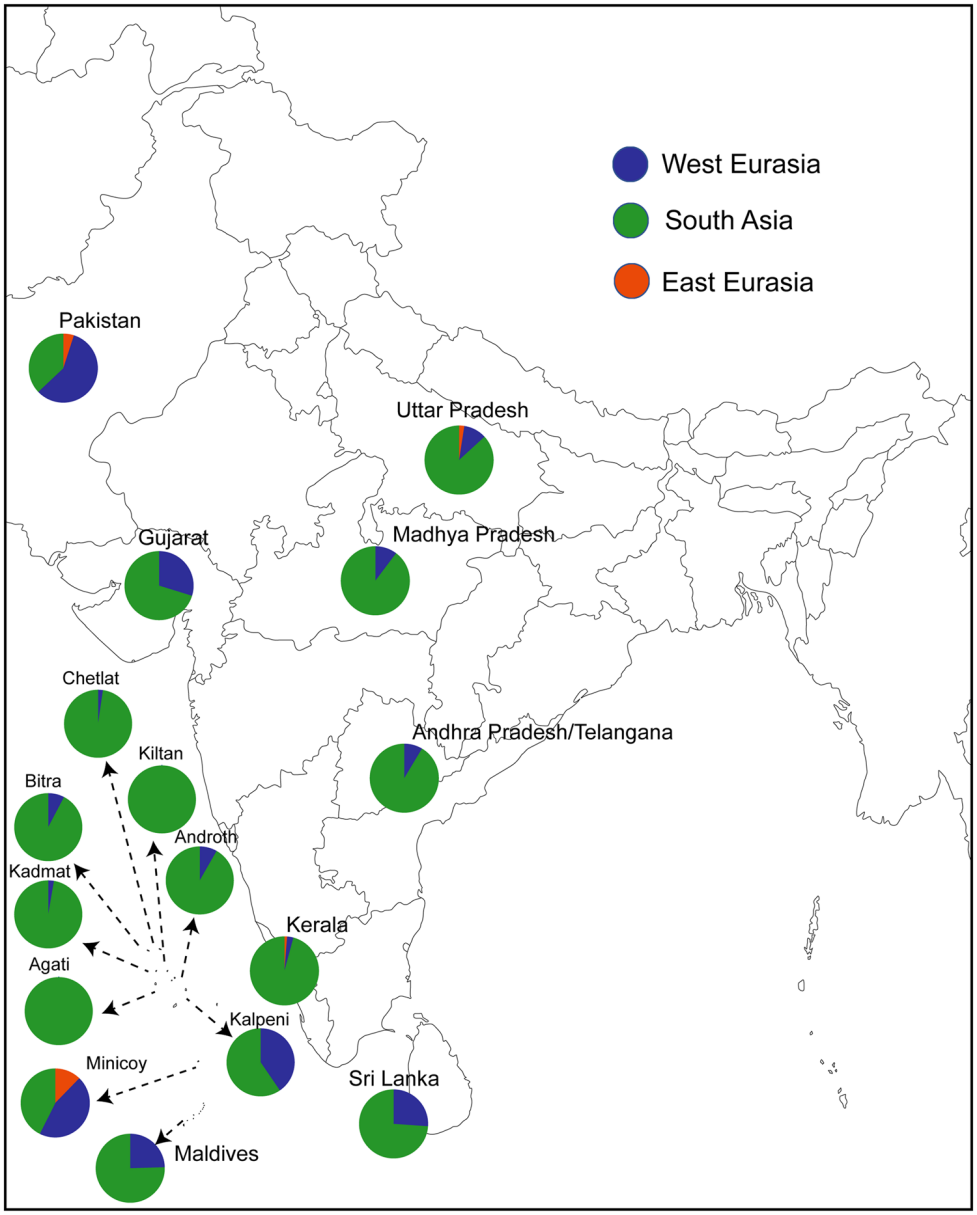
The spatial distribution of regional maternal components in Lakshadweep islanders in comparison with other neighbouring populations. South Asian haplogroups—mtDNA: M2–6, N5, M33–65, R5–8, and R31–32. East Eurasian haplogroups—A–G, M7–12, M66, R22, and N9. West Eurasian haplogroups H-K, U1, U4, U7 and U9 Unresolved haplogroups—M*, R*, N* including other lineages, for example, M31 and M32.

Our analysis on mtDNA has revealed that nearly 56% of the maternal lineages of Lakshadweep islands belong to three major haplogroups (M2b2, M30 and R30) (Table 1 and Supplementary Fig. 2). It is interesting to note that in the background of major haplogroups, all of the individuals carried mostly a single haplotype; therefore we call them as founder haplogroup (Supplementary Table 1). These founder haplogroups are virtually present in all the studied islands. Notably, haplogroup R30, which is also reported previously in the coastal South Asia including Sri Lanka^21,30^, is the most frequent haplogroup in many of the Lakshadweep islands (Table 1 and Supplementary Table 1). To have a spatial understanding of various maternal lineages present in Lakshadweep, we have grouped haplogroups in to regions and plotted with their geographical co-ordinates (Fig. 1). All the islands (except Minicoy) showed major component of maternal ancestry associated with South Asia. The southern most islands Kalpeni, and Minicoy show substantial West Eurasian-specific maternal ancestry, which is largely similar to the Maldives and Sri Lanka. Additionally, Minicoy island carried East Eurasian-specific haplogroups, which is otherwise virtually absent in this region.

**Figure 2:**
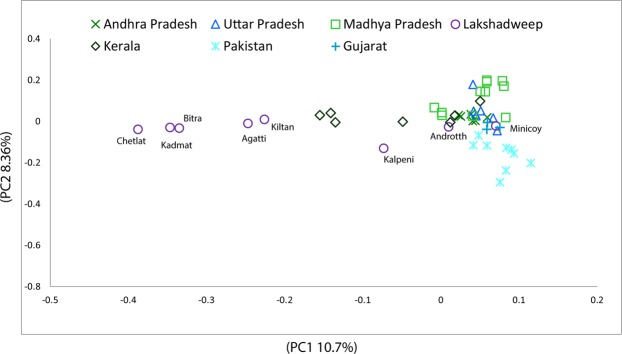
The scatter pattern of Lakshadweep islands groups in the PC1 vs. PC2 analysis for mtDNA within other regional South Asian populations.

The principle component analysis (PCA) of maternal ancestry including South Asian major regions formed a distinct cline of Lakshadweep populations, which is likely due to drift created by strong founder effect seen in their maternal ancestry (Fig. 2). The closest cluster to many of these islands are the populations of Kerala with whom they share their most common haplogroups i.e. M2 and R30 (Supplementary Table 2).

We measured the haplotype diversity of island populations with respect to the mainland South Asian populations (Supplementary Fig. 2a). Our data indicate a significant (two tailed p value < 0.001) reduction in the haplotype diversity of Lakshadweep populations. The most likely explanation of this reduction is that a few individuals were introduced to these islands, which therefore created a ‘bottleneck followed by founder effect’ like scenario.

**Figure 3:**
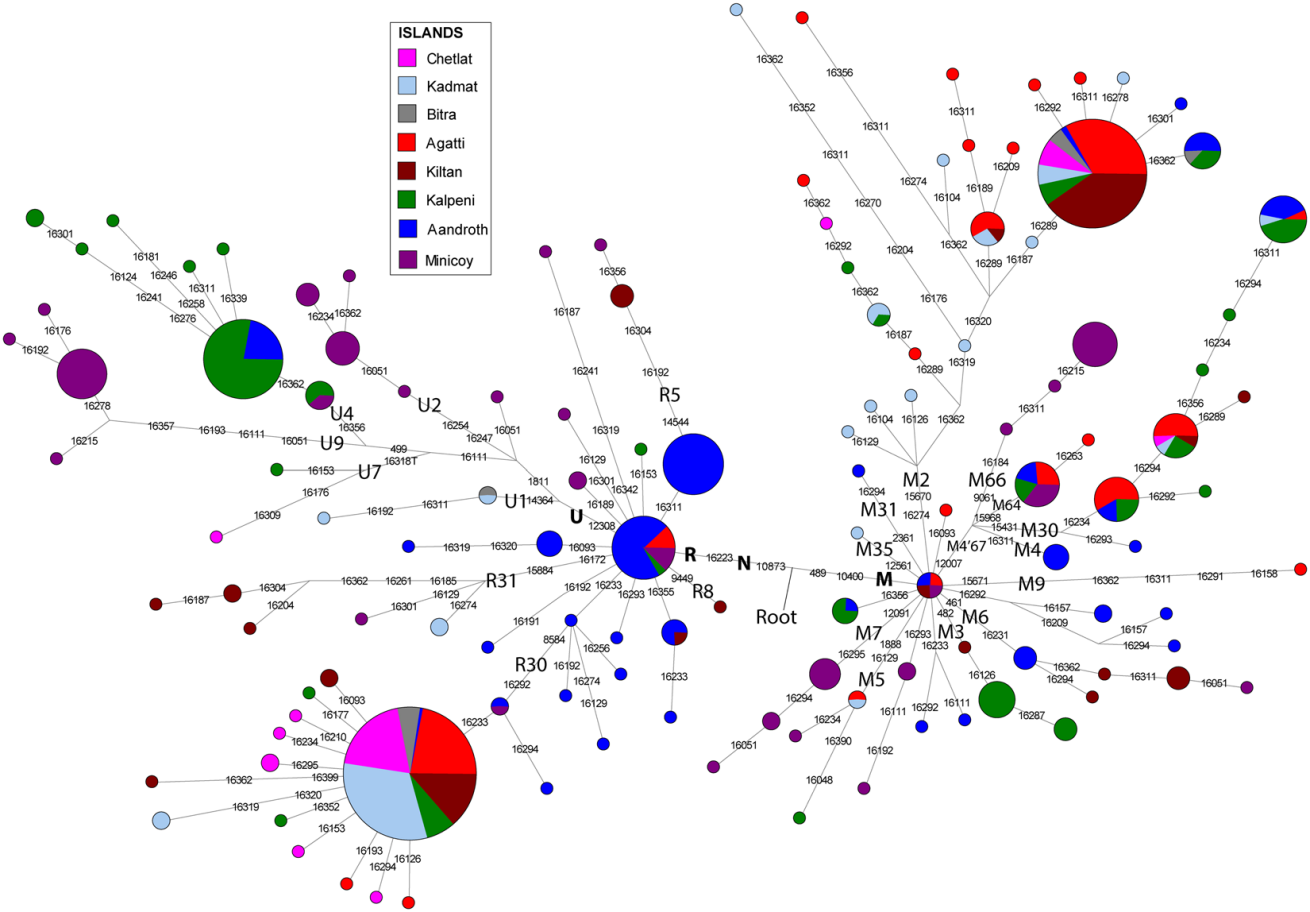
The median-joining network of 557 mtDNAs belonging to Lakshadweep islands. Each sample represented on the diagram has been sequenced for the HVS-I region and genotyped for the coding region mutations that are indicated in Supplementary Table 1. Circle sizes are proportional to the number of mtDNAs with that haplotype.

In order to have better understanding of founder lineages present in different islands of Lakshadweep we have reconstructed a median-joining network of maternal haplogroups identified for Lakshadweep islands (Fig. 3). The network analysis clearly showed the high prevalence of specific haplotypes present in the islands, likely stemming out from a single founder in each of their haplogroup background. Apart from major common founder maternal lineages, such as hg R30 followed by hg M2 (the top two maternal founder lineages of several islands), some of the islands carry specific founder lineages. For example, island Minicoy carry founder haplogroups M7 and M66 (East Eurasian specific) as well as hg U9 (West Eurasian-specific). Similarly, island Kalpeni harbour hg U4 (West Eurasian-specific) (Fig. 3 and Supplementary Fig. 1). The sharing of many of the maternal lineages among islands of Lakshadweep advocates a high level of intra-island maternal gene flow. At the inter-population level, they are closest to the Kerala populations with whom they share the same tongue (Supplementary Table 2). We have performed AMOVA analysis to compare the genetic relatedness of the Lakshadweep with the mainland South Asian populations. The analysis suggested a close genetic affinity of Lakshadweep populations with the South Indian Kerala populations (Table 2).

**Table 2:** The population differentiation analysis by AMOVA comparing Lakshadweep populations with the other South Asian mainland and island populations.

Population	Among Populations	Within Populations	*F*st	Among Populations	Within Populations	*F*st
	mtDNA			Y chromosome		
Pakistan and West India	8.46	91.54	0.08459	10.2	89.8	0.10197
North India	7.58	92.42	0.07578	8.09	91.91	0.0809
Andhra Pradesh/Telangana	7.34	92.66	0.07336	14.93	85.07	0.1492
Kerala	6.68	93.32	0.06685	35.46	64.54	0.35461
Sri Lanka	7.99	92.01	0.07064	13.3	86.7	0.13304
the Maldives	—	—	—	6.71	93.29	0.07156

p value < 0.05.

**Figure 4:**
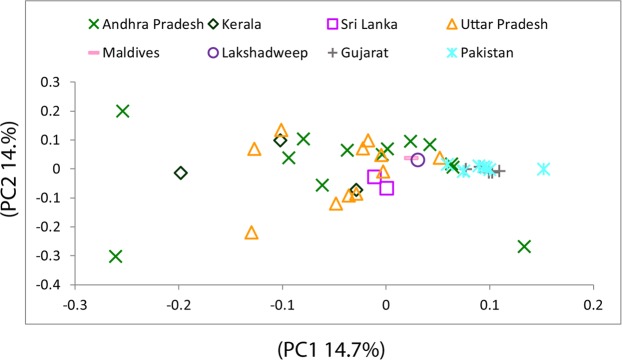
The genetic affinity of Lakshadweep island populations with other regional South Asian populations in the PC1 vs. PC2 analysis obtained from haplogroup frequencies for the Y chromosome.

**Figure 5:**
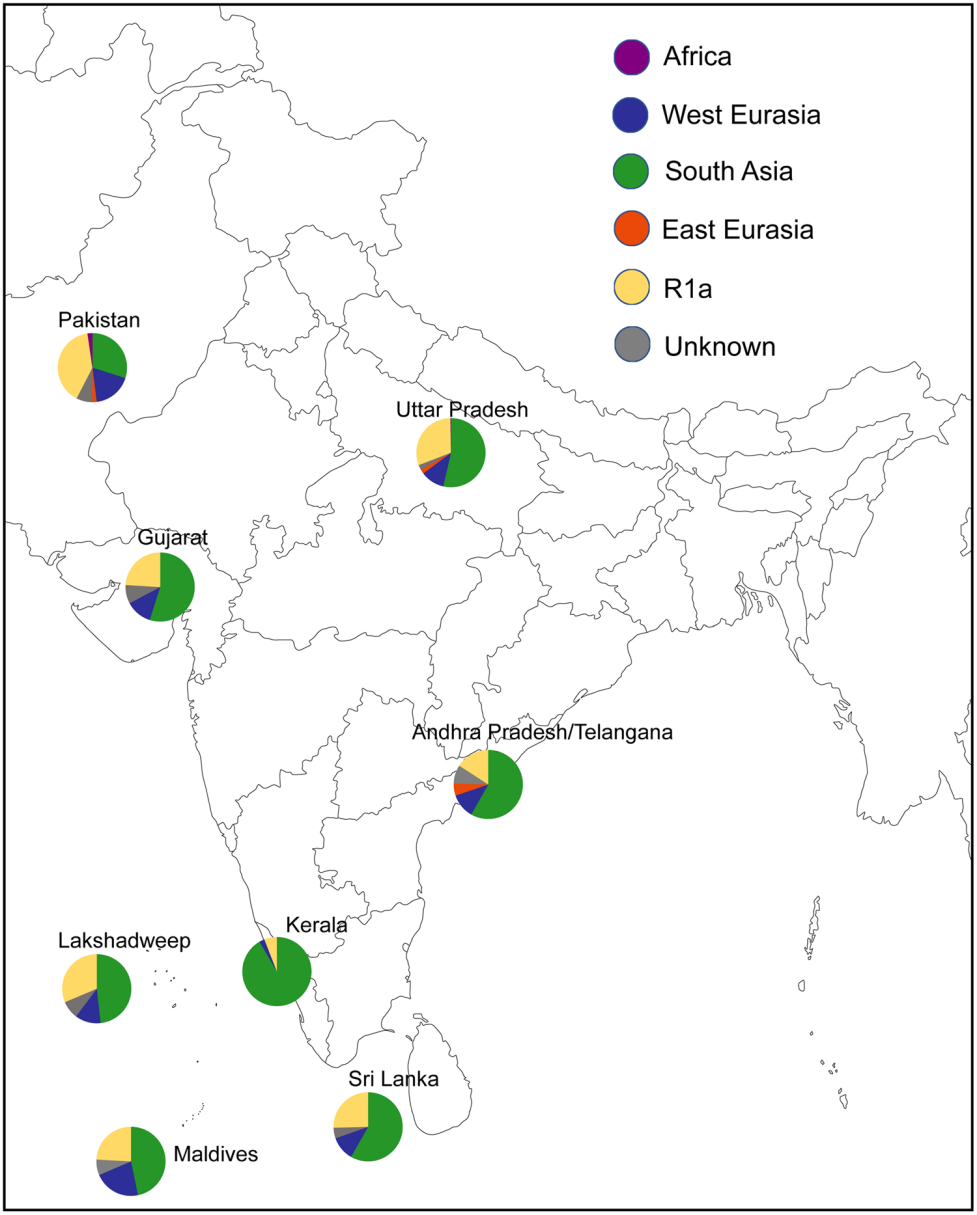
The spatial distribution of regional paternal components in Lakshadweep islanders in comparison with other neighbouring populations. South Asian haplogroups—C5, F, H, L, and R2. Southeast Asian haplogroups—C2, C3, D, and M–O. West Eurasian haplogroups G,I, and J. Unresolved haplogroups—K, P, Q and R1a.

In order to assess the paternal ancestry, we further analysed the Y chromosome markers among 166 individuals of Lakshadweep (Supplementary Tables 2 and 3). Since the sample numbers genotyped for Y chromosomal markers from various islands were uneven and five out of nine islands were genotyped for less than 20 individuals, we have pooled all the Y chromosomal data and represented as single data of Lakshadweep (Supplementary Tables 2 and 3). The paternal affinity illustrates a distinct pattern in comparison with maternal ancestry. In the PCA analysis, Lakshadweep inclines towards Maldives population (Fig. 4), which is likely due to hg J2-M172. In the region wise comparison, the paternal ancestry components of Lakshadweep are more similar to the Maldives, North India and Pakistan rather than Kerala (Fig. 5 and Table 2). This suggests a gene flow to Lakshadweep from all of these regions. However, similar to maternal founders, we also see a reduction in haplotype diversity due to strong founder effect for paternal lineages (Supplementary Fig. 3b). Three haplogroups J2a-M172, Ra1-M17 and R2a-M124 harbour approximately 85% of the paternal ancestry of the Lakshadweep. The most frequent haplogroup was hg R2a-M124 followed by hg R1a1-M17 (Supplementary Table 2). In comparison to the mainland India, the Maldives and Sri Lanka, Lakshadweep show reduced paternal diversity and stronger founder effect (Supplementary Figs 1 and 3b). However, due to absence of Y-STR data, we could not determine if all the persons belonging to one Y chromosomal haplogroup, has been derived from a single founder haplotype or from a multiple founder haplotypes.

In conclusions, the Lakshadweep populations share a large number of ancestries within the islands (except Minicoy). In spite of the fact that these islands served as a maritime crossroad, we have observed a strong founder effect with reduced diversity for both maternal and paternal ancestries. The maternal ancestry of the islands is largely derived from Southern India, whereas the paternal ancestry is showing higher affinity with the populations of the Maldives or Northern or Western part of the South Asia. The most diverse island is Minicoy which might have served as a popular destination for maritime sailors, thus received gene flow from various directions.

## Supplementary information

The Supplementary file contains three each of figures and tables.
