# Supplementary material for: The peopling of Lakshadweep Archipelago
Source: Sci Rep. 2019 May 6;9:6968. doi: 10.1038/s41598-019-43384-3 (PMC6502849; doi:10.1038/s41598-019-43384-3)
Supplement: Supplementary file 1 — The Supplementary file contains three each of figures and tables. [file 41598_2019_43384_MOESM1_ESM.pdf]

Supplementary File

The peopling of Lakshadweep Archipelago

Mohammed S Mustak [1,2], Niraj Rai [3], Mohan Rao Naveen [1], Satya Prakash [2], S. Justin Carlus [2], Nagarjuna Pasupuleti [1], Anshika Srivastava [4], Prajjval Pratap Singh [4], Idrees Babu [5], Pavan Kumar Dubey [6], Gyaneshwer Chaubey [4,7] and Kumarasamy Thangaraj [2*]

[1]Department of Applied Zoology, Mangalore University, Mangalore 574199, India
[2] CSIR-Centre for Cellular and Molecular Biology, Uppal Road, Hyderabad, 500007, India
[3]Birbal Sahni Institute of Palaeosciences, 53 University Road, Lucknow, 226007, India
[4]Cytogenetics Laboratory, Department of Zoology, Banaras Hindu University, Varanasi, 221005, India
[5]Department of Science and Technology, Lakshadweep Administration, Kavaratti, 682555, India
[6]Prosthodontics Unit, Faculty of Dental Sciences, Institute of Medical Sciences, Varanasi, 221005, India
[7]Evolutionary Biology Group, Estonian Biocentre, Riia23b, 51010, Tartu, Estonia

MSM and NR: equal contribution
KT and GC: equal senior authors

Correspondence to:

Dr Kumarasamy Thangaraj
CSIR-Centre for Cellular and Molecular Biology, Uppal Road, Hyderabad, 500007, India
email: thangs@ccmb.res.in; Ph- +91-40-27182828; Fax : +91-40-27160591

The Supplementary file contains three each of figures and tables.

Supplementary Figure 1. The location of Lakshadweep Islands in the map.

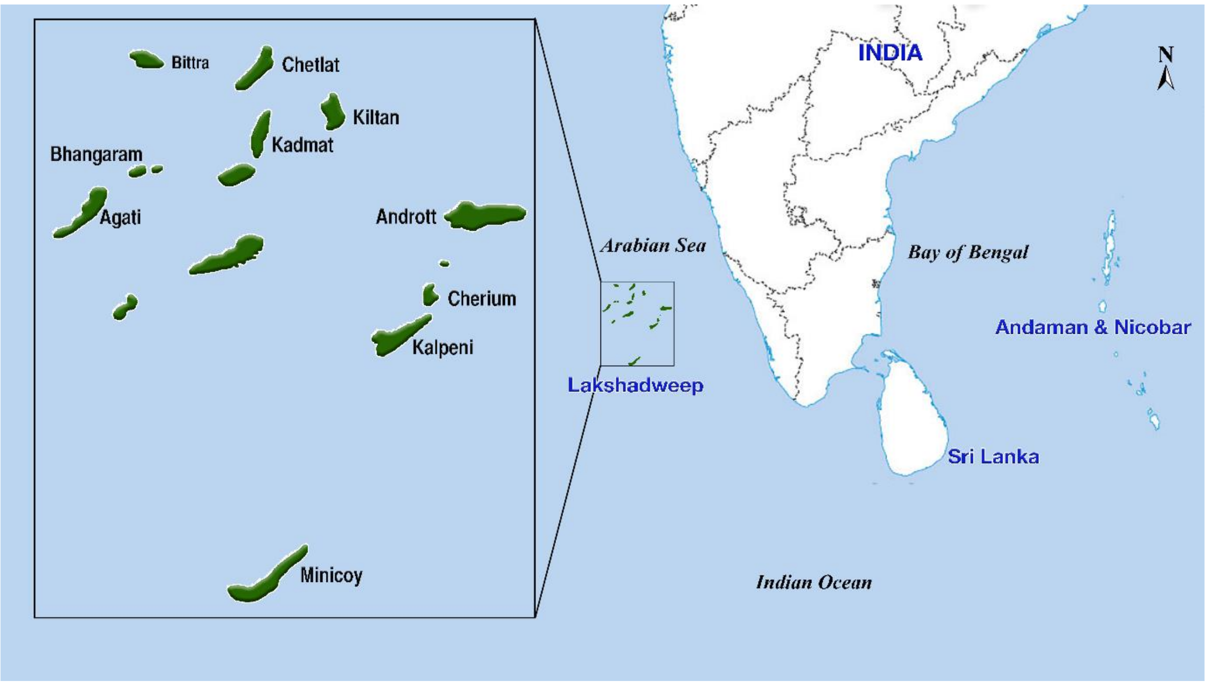

Supplementary Figure 2. Bar-plot depicting a) mtDNA b) Y chromosome frequencies of different haplogroup among studied populations with respect to other South Asian populations. The major haplogroups present in the Lakshadweep has been shown in bold letters.

a

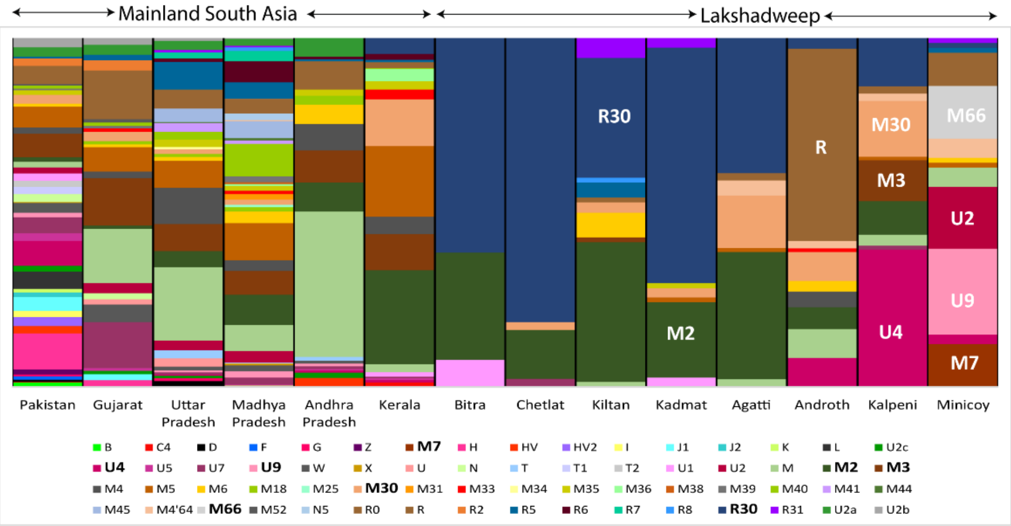

b

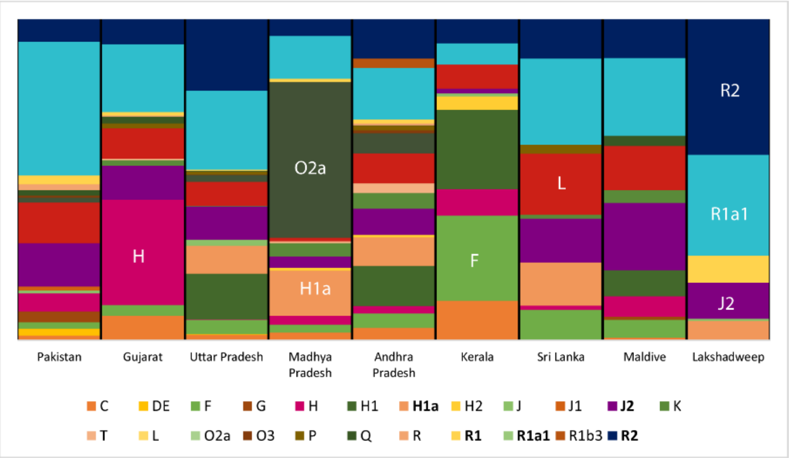

Supplementary Figure 3. The haplotype diversity of Lakshadweep populations (a mtDNA, b) Y chromosome. The substantial reduction of diversity suggest a ‘bottleneck followed by founder effect’ scenario.

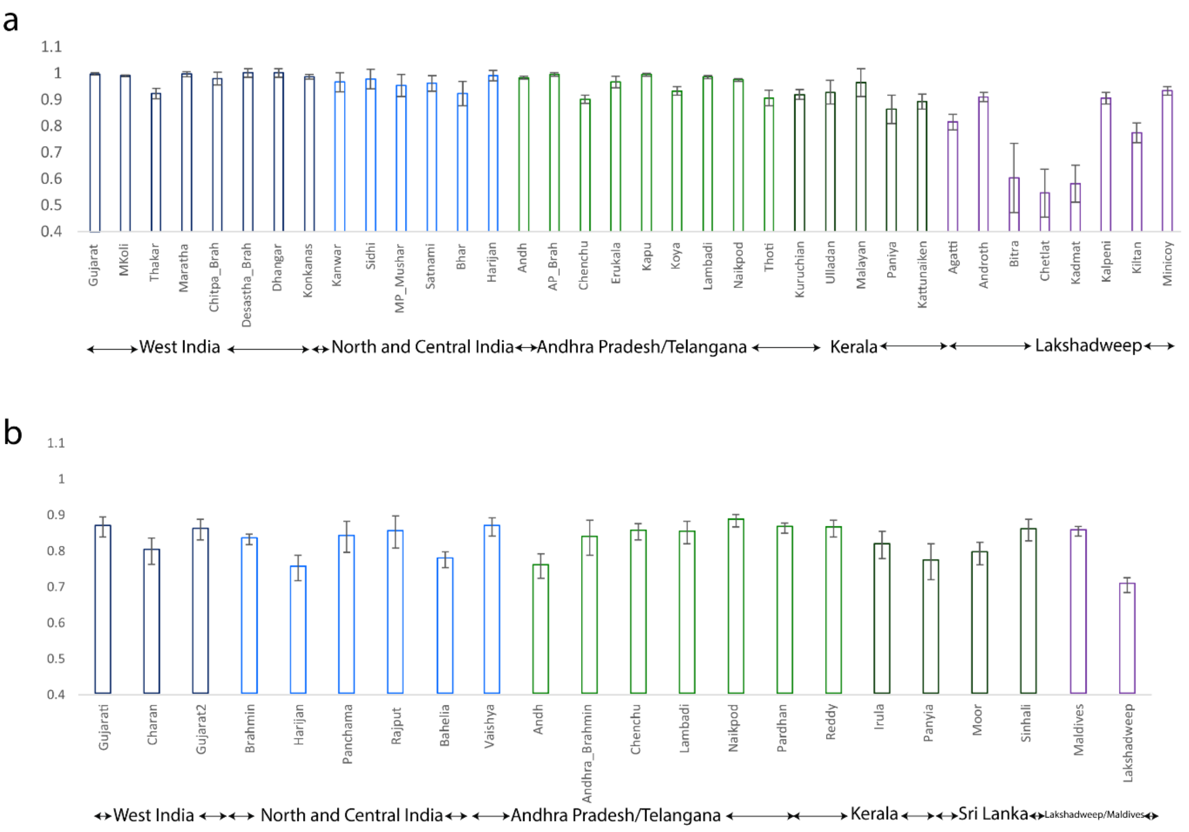

Supplementary File

Supplementary Table 1. The samplewise mtDNA HVS-I and coding region mutations observed in different islands of the Lakshadweep

Island	Sample Code	HVS-I	Coding region	Haplogroup
Agatti	AgY47	16223T 16497G	10398-10400-15043-15301	M
Agatti	AgZ25	16093C 16223T	10398-10400-15043-15301	M
Agatti	AgY44	16223T 16311C 16319A 16320T 16356C	447G-10398-10400-13254-15043-15301-15670	M2b
Agatti	AgX31	16223T 16274A 16289G 16319A 16320T 16362C	447G-10398-10400-13254-15043-15301-15670	M2b2
Agatti	AgZ3	16223T 16274A 16289G 16319A 16320T 16362C	447G-10398-10400-13254-15043-15301-15670	M2b2
Agatti	AgX3	16223T16274A 16289G 16319A 16320T 16362C	447G-10398-10400-13254-15043-15301-15670	M2b2
Agatti	AgY5	16187T 16223T 16274A 16289G 16292T 16362C	447G-10398-10400-13254-15043-15301-15670	M2b2
Agatti	AgX24	16187T 16223T 16274A 16289G 16319A 16320T 16362C	447G-10398-10400-13254-15043-15301-15670	M2b2
Agatti	AgX4	16187T 16223T 16274A 16289G 16319A 16320T 16362C	447G-10398-10400-13254-15043-15301-15670	M2b2
Agatti	AgX47	16187T 16223T 16274A 16289G 16319A 16320T 16362C	447G-10398-10400-13254-15043-15301-15670	M2b2
Agatti	AgX32	16187T 16223T 16274A 16289G 16319A 16320T 16362C	447G-10398-10400-13254-15043-15301-15670	M2b2
Agatti	AgX5	16187T 16223T 16274A 16289G 16319A 16320T 16362C	447G-10398-10400-13254-15043-15301-15670	M2b2
Agatti	AgX11	16187T 16223T 16274A 16289G 16319A 16320T 16362C	447G-10398-10400-13254-15043-15301-15670	M2b2
Agatti	AgX17	16187T 16223T 16274A 16289G 16319A 16320T 16362C	447G-10398-10400-13254-15043-15301-15670	M2b2
Agatti	AgX18	16187T 16223T 16274A 16289G 16319A 16320T 16362C	447G-10398-10400-13254-15043-15301-15670	M2b2
Agatti	AgY14	16187T 16223T 16274A 16289G 16319A 16320T 16362C	447G-10398-10400-13254-15043-15301-15670	M2b2
Agatti	AgY15	16187T 16223T 16274A 16289G 16319A 16320T 16362C	447G-10398-10400-13254-15043-15301-15670	M2b2
Agatti	AgY2	16187T 16223T 16274A 16289G 16319A 16320T 16362C	447G-10398-10400-13254-15043-15301-15670	M2b2
Agatti	AgY23	16187T 16223T 16274A 16289G 16319A 16320T 16362C	447G-10398-10400-13254-15043-15301-15670	M2b2
Agatti	AgY29	16187T 16223T 16274A 16289G 16319A 16320T 16362C	447G-10398-10400-13254-15043-15301-15670	M2b2
Agatti	AgY31	16187T 16223T 16274A 16289G 16319A 16320T 16362C	447G-10398-10400-13254-15043-15301-15670	M2b2
Agatti	AgY36	16187T 16223T 16274A 16289G 16319A 16320T 16362C	447G-10398-10400-13254-15043-15301-15670	M2b2
Agatti	AgY42	16187T 16223T 16274A 16289G 16319A 16320T 16362C	447G-10398-10400-13254-15043-15301-15670	M2b2
Agatti	AgZ17	16187T 16223T 16274A 16289G 16319A 16320T 16362C	447G-10398-10400-13254-15043-15301-15670	M2b2
Agatti	AgZ21	16187T 16223T 16274A 16289G 16319A 16320T 16362C	447G-10398-10400-13254-15043-15301-15670	M2b2
Agatti	AgZ22	16187T 16223T 16274A 16289G 16319A 16320T 16362C	447G-10398-10400-13254-15043-15301-15670	M2b2
Agatti	AgX12	16187T 16223T 16274A 16289G 16319A 16320T 16362C	447G-10398-10400-13254-15043-15301-15670	M2b2
Agatti	AgX15	16187T 16223T 16274A 16289G 16319A 16320T 16362C	447G-10398-10400-13254-15043-15301-15670	M2b2
Agatti	AgX10	16187T 16223T 16274A 16289G 16311C 16319A 16320T 16362C	447G-10398-10400-13254-15043-15301-15670	M2b2
Agatti	AgY43	16187T 16223T 16274A 1628916292T 16319A 16320T 16362C	447G-10398-10400-13254-15043-15301-15670	M2b2
Agatti	AgX9	16187T 16274A 16289G 16319A 16320T 16362C	447G-10398-10400-13254-15043-15301-15670	M2b2
Agatti	AgX25	16223T 16223T 16274A 16289G 16319A 16320T 16362C	447G-10398-10400-13254-15043-15301-15670	M2b2
Agatti	AgZ24	16223T 16274A 16289G 16362C	447G-10398-10400-13254-15043-15301-15670	M2b2
Agatti	AgX33	16189C 16223T 16274A 16289G 16319A 16320T 16362C	447G-10398-10400-13254-15043-15301-15670	M2b2
Agatti	AgX35	16189C 16223T 16274A 16289G 16311C 16319A 16320T 16362C	447G-10398-10400-13254-15043-15301-15670	M2b2
Agatti	AgX39	16209C 16223T 16274A 16289G 16319A 16320T 16362C	447G-10398-10400-13254-15043-15301-15670	M2b2
Agatti	AgZ4(Reblank)	16223T 16234T	195A-10398-10400-15043-15301-15431	M30
Agatti	AgY35	16223T 16234T 16294T	195A-10398-10400-15043-15301-15431	M30

Supplementary File

Agatti	AgY37	16223T 16234T 16294T	195A-10398-10400-15043-15301-15431	M30
Agatti	AgZ20	16223T 16234T 16294T	195A-10398-10400-15043-15301-15431	M30
Agatti	AgZ11	16223T 16234T 16294T	195A-10398-10400-15043-15301-15431	M30
Agatti	AgX48	16223T 16234T 16294T 16497G	195A-10398-10400-15043-15301-15431	M30
Agatti	AgX29	16223T 16234T	195A-10398-10400-15043-15301-15431	M30
Agatti	AgX30	16223T 16234T	195A-10398-10400-15043-15301-15431	M30
Agatti	AgX49	16223T 16234T	195A-10398-10400-15043-15301-15431	M30
Agatti	AgY6	16223T 16234T	195A-10398-10400-15043-15301-15431	M30
Agatti	AgZ23	16223T 16234T	195A-10398-10400-15043-15301-15431	M30
Agatti	AgY17	16223T 16234T 16294T	195A-10398-10400-15043-15301-15431	M30
Agatti	AgX41	16223T 16311C 16356C	195A-10398-10400-15043-15301-15431	M30
Agatti	AgX20	16234T	195A-10398-10400-15043-15301-15431	M30
Agatti	AgX50	16129A 16223T	1888-10398-10400-15043-15301	M5
Agatti	AgY40	16223T	10398-10400-15043-15301-15968	M64
Agatti	AgX27	16223T	10398-10400-15043-15301-15968	M64
Agatti	AgZ15	16223T	10398-10400-15043-15301-15968	M64
Agatti	AgZ18	16223T 16263C	10398-10400-15043-15301-15968	M64
Agatti	AgX1	126086C 16158G 16223T 16291T 16311C 16362C	10398-10400-15043-15301-15671	M9a1b1
Agatti	AgX42	rCRS	15326	R
Agatti	AgY46	rCRS	15326	R
Agatti	AgZ27	16193T 16292T 16497G	8584-10577-15326	R30
Agatti	AgZ10	16292T	8584-10577-15326	R30
Agatti	AgX28	16292T 16497G	8584-10577-15326	R30
Agatti	AgX36	16292T 16497G	8584-10577-15326	R30
Agatti	AgX45	16292T 16497G	8584-10577-15326	R30
Agatti	AgX46	16292T 16497G	8584-10577-15326	R30
Agatti	AgY1	16292T 16497G	8584-10577-15326	R30
Agatti	AgY18	16292T 16497G	8584-10577-15326	R30
Agatti	AgY19	16292T 16497G	8584-10577-15326	R30
Agatti	AgY20	16292T 16497G	8584-10577-15326	R30
Agatti	AgY24	16292T 16497G	8584-10577-15326	R30
Agatti	AgY41	16292T 16497G	8584-10577-15326	R30
Agatti	AgY51	16292T 16497G	8584-10577-15326	R30
Agatti	AgY7	16292T 16497G	8584-10577-15326	R30
Agatti	AgZ19	16292T 16497G	8584-10577-15326	R30
Agatti	AgZ26	16292T 16497G	8584-10577-15326	R30
Agatti	AgX23	16292T 16497G	8584-10577-15326	R30
Agatti	AgY10	16292T 16497G	8584-10577-15326	R30
Agatti	AgY11	16292T 16497G	8584-10577-15326	R30
Agatti	AgY21	16292T 16497G	8584-10577-15326	R30
Agatti	AgY22	16292T 16497G	8584-10577-15326	R30
Agatti	AgY30	16292T 16497G	8584-10577-15326	R30
Agatti	AgY38	16292T 16497G	8584-10577-15326	R30
Agatti	AgY45	16292T 16497G	8584-10577-15326	R30
Agatti	AgY48	16292T 16497G	8584-10577-15326	R30
Agatti	AgY49	16292T 16497G	8584-10577-15326	R30
Agatti	AgY8	16292T 16497G	8584-10577-15326	R30
Agatti	AgZ1	16292T 16497G	8584-10577-15326	R30

Supplementary File

Agatti	AgZ12	16292T 16497G	8584-10577-15326	R30
Agatti	AgZ13	16292T 16497G	8584-10577-15326	R30
Agatti	AgZ2	16292T 16497G	8584-10577-15326	R30
Agatti	AgZ28	16292T 16497G	8584-10577-15326	R30
Agatti	AgZ5	16292T 16497G	8584-10577-15326	R30
Agatti	AgZ8	16292T 16497G	8584-10577-15326	R30
Agatti	AgZ9	16292T 16497G	8584-10577-15326	R30
Agatti	AgX40	16126C 16292T 16497G	8584-10577-15326	R30
Androth	ANb43	16111T 16233T	10398-10400-15043-15301	M
Androth	M100	16223T	10398-10400-15043-15301	M
Androth	ANa48	16223T 16356C	10398-10400-15043-15301	M
Androth	ANb47	16233T 16292T	10398-10400-15043-15301	M
Androth	Anb10	16157C 16292T	10398-10400-15043-15301	M
Androth	Anb8	16157C 16292T	10398-10400-15043-15301	M
Androth	Anb17	16157C 16209C 16223T 16292T	10398-10400-15043-15301	M
Androth	Anb20	16209C 16223T 16292T 16294T	10398-10400-15043-15301	M
Androth	ANa7	16187T 16223T 16274A 16289G 16301T 16319A 16320T 16362C	447G-10398-10400-13254-15043-15301-15670	M2b2
Androth	ANc46	16187T 16223T 16274A 16289G 16319A 16320T 16362C	447G-10398-10400-13254-15043-15301-15670	M2b2
Androth	ANc17	16187T 16274A 16289G 16289G 16319A 16320T	447G-10398-10400-13254-15043-15301-15670	M2b2
Androth	ANc36	16187T 16274A 16289G 16289G 16319A 16320T	447G-10398-10400-13254-15043-15301-15670	M2b2
Androth	ANc37	16187T 16274A 16289G 16289G 16319A 16320T	447G-10398-10400-13254-15043-15301-15670	M2b2
Androth	ANc3	16187T 16274A 16289G 16289G 16319A 16320T	447G-10398-10400-13254-15043-15301-15670	M2b2
Androth	Anb19	16223T 16293G	195A-10398-10400-15043-15301-15431	M30
Androth	ANa10	16223T 16311C 16356C	195A-10398-10400-15043-15301-15431	M30
Androth	ANa15	16223T 16311C 16356C	195A-10398-10400-15043-15301-15431	M30
Androth	ANa36	16223T 16311C 16356C	195A-10398-10400-15043-15301-15431	M30
Androth	ANc50	16223T 16311C 16356C	195A-10398-10400-15043-15301-15431	M30
Androth	ANa44	16223T 16311C 16356C	195A-10398-10400-15043-15301-15431	M30
Androth	ANc11	16234T	195A-10398-10400-15043-15301-15431	M30
Androth	ANc15	16234T	195A-10398-10400-15043-15301-15431	M30
Androth	ANa37	16223T 16294T	2361-10398-10400-15043-15301	M33a1a
Androth	ANa39	16223T 16311C	10398-10400-12007-15043-15301	M4
Androth	ANa8	16223T 16311C	10398-10400-12007-15043-15301	M4
Androth	ANa9	16223T 16311C	10398-10400-12007-15043-15301	M4
Androth	ANb29	16223T 16311C	10398-10400-12007-15043-15301	M4
Androth	ANc47	16223T	10398-10400-15043-15301-15968	M64
Androth	ANc49	16223T	10398-10400-15043-15301-15968	M64
Androth	ANc26	16231C	461-10398-10400-15043-15301	M6a
Androth	ANc28	16231C	461-10398-10400-15043-15301	M6a
Androth	ANc6	16231C	461-10398-10400-15043-15301	M6a
Androth	ANc39	16355T	15326	R
Androth	ANc18	16355T	15326	R
Androth	ANc22	16355T	15326	R
Androth	Anb3	16293G	15326	R
Androth	Anb21	16192T 16191T	15326	R
Androth	ANc4	16192T 16233T	15326	R
Androth	ANc32	16233T	15326	R

Supplementary File

Androth	ANc30	16233T 16256T	15326	R
Androth	ANc2	16233T 16355T	15326	R
Androth	ANc19	16129A 16233T 16274A	15326	R
Androth	ANa38	rCRS	15326	R
Androth	Anb4	rCRS	15326	R
Androth	Anb5	rCRS	15326	R
Androth	Anb7	rCRS	15326	R
Androth	Anb9	rCRS	15326	R
Androth	ANc12	rCRS	15326	R
Androth	ANc13	rCRS	15326	R
Androth	ANc23	rCRS	15326	R
Androth	ANc24	rCRS	15326	R
Androth	ANc25	rCRS	15326	R
Androth	ANc29	rCRS	15326	R
Androth	ANc31	rCRS	15326	R
Androth	ANc33	rCRS	15326	R
Androth	ANc35	rCRS	15326	R
Androth	ANc38	rCRS	15326	R
Androth	ANc40	rCRS	15326	R
Androth	ANc7	rCRS	15326	R
Androth	ANc1	16093C	15326	R
Androth	ANc45	16093C	15326	R
Androth	ANc8	16093C	15326	R
Androth	ANc9	16093C	15326	R
Androth	ANc5	16093C 16319A 16320T	15326	R
Androth	ANa1	16311C	15326	R
Androth	ANa11	16311C	15326	R
Androth	ANa12	16311C	15326	R
Androth	ANa13	16311C	15326	R
Androth	ANa17	16311C	15326	R
Androth	ANa19	16311C	15326	R
Androth	ANa2	16311C	15326	R
Androth	ANa20	16311C	15326	R
Androth	ANa21	16311C	15326	R
Androth	ANa24	16311C	15326	R
Androth	ANa26	16311C	15326	R
Androth	ANa3	16311C	15326	R
Androth	ANa34	16311C	15326	R
Androth	ANa35	16311C	15326	R
Androth	ANa4	16311C	15326	R
Androth	ANa45	16311C	15326	R
Androth	ANa46	16311C	15326	R
Androth	ANa5	16311C	15326	R
Androth	ANa6	16311C	15326	R
Androth	ANb13	16311C	15326	R
Androth	ANa47	16311C	15326	R
Androth	ANc21	16233T 16292T 16497G	8584-10577-15326	R30
Androth	ANc10	16233T 16292T 16294T 16497G	8584-10577-15326	R30

Supplementary File

Androth	ANa14	16292T 16497G	8584-10577-15326	R30
Androth	Anb2	16111T 16356C 16362C	499-1811-12308-12372-15326-15693	U4
Androth	ANa23	16111T 16356C 16362C	499-1811-12308-12372-15326-15693	U4
Androth	ANa25	16111T 16356C 16362C	499-1811-12308-12372-15326-15693	U4
Androth	ANa40	16111T 16356C 16362C	499-1811-12308-12372-15326-15693	U4
Androth	ANa41	16111T 16356C 16362C	499-1811-12308-12372-15326-15693	U4
Androth	ANa42	16111T 16356C 16362C	499-1811-12308-12372-15326-15693	U4
Androth	ANa43	16111T 16356C 16362C	499-1811-12308-12372-15326-15693	U4
Androth	Anb1	16111T 16356C 16362C	499-1811-12308-12372-15326-15693	U4
Bitra	LB1	16187T 16274A 16289G 16319A 16320T	447G-10398-10400-13254-15043-15301-15670	M2b2
Bitra	LB3	16187T 16274A 16289G 16319A 16320T 16362C	447G-10398-10400-13254-15043-15301-15670	M2b2
Bitra	Bi14	16187T 16274A 16289G 16319A 16320T 16362C	447G-10398-10400-13254-15043-15301-15670	M2b2
Bitra	LB13	16187T 16274A 16289G 16319A 16320T 16362C	447G-10398-10400-13254-15043-15301-15670	M2b2
Bitra	LB10	16292T 16497G	8584-10577-15326	R30
Bitra	LB11	16292T 16497G	8584-10577-15326	R30
Bitra	LB12	16292T 16497G	8584-10577-15326	R30
Bitra	LB2	16292T 16497G	8584-10577-15326	R30
Bitra	LB4	16292T 16497G	8584-10577-15326	R30
Bitra	LB6	16292T 16497G	8584-10577-15326	R30
Bitra	LB7	16292T 16497G	8584-10577-15326	R30
Bitra	LB8	16292T 16497G	8584-10577-15326	R30
Bitra	LB9	16497G	12308-12372-14364-15326	U1a1
Chetlat	L23	16187T 16223T 16274A 16289G 16292T 16497G 16519C	447G-10398-10400-13254-15043-15301-15670	M2b2
Chetlat	LC10	16187T 16223T 16274A 16289G 16319A 16320T 16362C	447G-10398-10400-13254-15043-15301-15670	M2b2
Chetlat	L27	16187T 16223T 16274A 16289G 16319A 16320T 16362C 16519C	447G-10398-10400-13254-15043-15301-15670	M2b2
Chetlat	L39	16187T 16274A 16289G 16319A 16320T 16362C	447G-10398-10400-13254-15043-15301-15670	M2b2
Chetlat	L45	16187T 16274A 16289G 16319A 16320T 16362C	447G-10398-10400-13254-15043-15301-15670	M2b2
Chetlat	L46	16187T 16274A 16289G 16319A 16320T 16362C	447G-10398-10400-13254-15043-15301-15670	M2b2
Chetlat	LC1	16223T 16234T 16294T 16519C	195A-10398-10400-15043-15301-15431	M30
Chetlat	LC17	16153A 16292T 16497G 16519C	8584-10577-15326	R30
Chetlat	LC14	16210G 16292T 16465T 16497G 16519C	8584-10577-15326	R30
Chetlat	L30	16234T 16292T	8584-10577-15326	R30
Chetlat	L36	16292T 16294T	8584-10577-15326	R30
Chetlat	LC12	16292T 16295T 16497G 16519C	8584-10577-15326	R30
Chetlat	LC7	16292T 16295T 16497G 16519C	8584-10577-15326	R30
Chetlat	L31	16292T 16497G	8584-10577-15326	R30
Chetlat	L32	16292T 16497G	8584-10577-15326	R30
Chetlat	L33	16292T 16497G	8584-10577-15326	R30
Chetlat	L34	16292T 16497G	8584-10577-15326	R30
Chetlat	L35	16292T 16497G	8584-10577-15326	R30
Chetlat	L37	16292T 16497G	8584-10577-15326	R30
Chetlat	L38	16292T 16497G	8584-10577-15326	R30
Chetlat	L41	16292T 16497G	8584-10577-15326	R30
Chetlat	L42	16292T 16497G	8584-10577-15326	R30
Chetlat	L48	16292T 16497G	8584-10577-15326	R30
Chetlat	L49	16292T 16497G	8584-10577-15326	R30

Supplementary File

Chetlat	LC50	16292T 16497G	8584-10577-15326	R30
Chetlat	L2	16292T 16497G 16519C	8584-10577-15326	R30
Chetlat	L21	16292T 16497G 16519C	8584-10577-15326	R30
Chetlat	L22	16292T 16497G 16519C	8584-10577-15326	R30
Chetlat	L24	16292T 16497G 16519C	8584-10577-15326	R30
Chetlat	L25	16292T 16497G 16519C	8584-10577-15326	R30
Chetlat	L26	16292T 16497G 16519C	8584-10577-15326	R30
Chetlat	L4	16292T 16497G 16519C	8584-10577-15326	R30
Chetlat	LC11	16292T 16497G 16519C	8584-10577-15326	R30
Chetlat	LC13	16292T 16497G 16519C	8584-10577-15326	R30
Chetlat	LC15	16292T 16497G 16519C	8584-10577-15326	R30
Chetlat	LC16	16292T 16497G 16519C	8584-10577-15326	R30
Chetlat	LC18	16292T 16497G 16519C	8584-10577-15326	R30
Chetlat	LC19	16292T 16497G 16519C	8584-10577-15326	R30
Chetlat	LC5	16292T 16497G 16519C	8584-10577-15326	R30
Chetlat	LC6	16292T 16497G 16519C	8584-10577-15326	R30
Chetlat	LC8	16292T 16497G 16519C	8584-10577-15326	R30
Chetlat	LC9	16292T 16497G 16519C	8584-10577-15326	R30
Chetlat	L44	16176T 16309G 16318t	1811-12308-12372-14569-15326	U7
Kadmat	LKd40	16104T 16274A 16319A 16320T	447G-9758-10398-10400-15043-15301-15670	M2a1
Kadmat	LKd51	16176T 16204T 16270T 16274A 16311C 16319A 16352C 16519C	447G-9758-10398-10400-15043-15301-15670	M2a1
Kadmat	LKd5	16223T 16274A 16319A 16362C 16527T	447G-9758-10398-10400-15043-15301-15670	M2a1
Kadmat	LKd52	16187T 16223T 16274A 16289G 16319A 16320T 16362C	447G-10398-10400-13254-15043-15301-15670	M2b2
Kadmat	LKd82	16187T 16223T 16274A 16278T 16289G 16319A 16320T 16362C	447G-10398-10400-13254-15043-15301-15670	M2b2
Kadmat	LKd56	16187T 16223T 16274A 16289G 16319A 16320T 16362C	447G-10398-10400-13254-15043-15301-15670	M2b2
Kadmat	LKd86	16187T 16223T 16274A 16289G 16319A 16320T 16362C	447G-10398-10400-13254-15043-15301-15670	M2b2
Kadmat	LKd62	16187T 16223T 16274A 16289G 16362C	447G-10398-10400-13254-15043-15301-15670	M2b2
Kadmat	LKd60	16187T 16223T 16274A 16289G 16362C 16519C	447G-10398-10400-13254-15043-15301-15670	M2b2
Kadmat	LKd32	16187T 16223T 16274A 16319A 16320T 16362C 16497G	447G-10398-10400-13254-15043-15301-15670	M2b2
Kadmat	LKd47	16187T 16274A 16289G 16319A 16320T 16362C	447G-10398-10400-13254-15043-15301-15670	M2b2
Kadmat	LKd8	16223T 16274A 16289G 16319A 16320T 16362C	447G-10398-10400-13254-15043-15301-15670	M2b2
Kadmat	LKd74	16223T 16274A 16289G 16319A 16320T 16362C	447G-10398-10400-13254-15043-15301-15670	M2b2
Kadmat	LKd20	16104T 16274A 16519C	447G-1598-10398-10400-15043-15301-15670	M2c
Kadmat	LKd67	16126C 16223T 16274A 16519C	447G-1598-10398-10400-15043-15301-15670	M2c
Kadmat	LKd27	16129A 16274A 16519C	447G-1598-10398-10400-15043-15301-15670	M2c
Kadmat	LKd76	16223T 16234T 16294T 16519C	195A-10398-10400-15043-15301-15431	M30
Kadmat	LKd84	16223T 16311C 16356C 16519C	195A-10398-10400-15043-15301-15431	M30
Kadmat	LKd24	16223T	10398-10400-12561-15043-15301	M35
Kadmat	LKd10	16129A 16223T	1888-10398-10400-15043-15301	M5
Kadmat	LKd37	16292T	8584-10577-15326	R30
Kadmat	LKd7	16292T	8584-10577-15326	R30
Kadmat	LKd9	16292T	8584-10577-15326	R30
Kadmat	LKd61	16292T 16319A 16320T 16497G 16519C	8584-10577-15326	R30
Kadmat	LKd63	16292T 16319A 16320T 16497G 16519C	8584-10577-15326	R30
Kadmat	LKd1	16292T 16497G	8584-10577-15326	R30

Supplementary File

Kadmat	LKd2	16292T 16497G	8584-10577-15326	R30
Kadmat	LKd3	16292T 16497G	8584-10577-15326	R30
Kadmat	LKd30	16292T 16497G	8584-10577-15326	R30
Kadmat	LKd4	16292T 16497G	8584-10577-15326	R30
Kadmat	LKd11	16292T 16497G 16519C	8584-10577-15326	R30
Kadmat	LKd12	16292T 16497G 16519C	8584-10577-15326	R30
Kadmat	LKd13	16292T 16497G 16519C	8584-10577-15326	R30
Kadmat	LKd15	16292T 16497G 16519C	8584-10577-15326	R30
Kadmat	LKd18	16292T 16497G 16519C	8584-10577-15326	R30
Kadmat	LKd21	16292T 16497G 16519C	8584-10577-15326	R30
Kadmat	LKd22	16292T 16497G 16519C	8584-10577-15326	R30
Kadmat	LKd23	16292T 16497G 16519C	8584-10577-15326	R30
Kadmat	LKd25	16292T 16497G 16519C	8584-10577-15326	R30
Kadmat	LKd26	16292T 16497G 16519C	8584-10577-15326	R30
Kadmat	LKd28	16292T 16497G 16519C	8584-10577-15326	R30
Kadmat	LKd29	16292T 16497G 16519C	8584-10577-15326	R30
Kadmat	LKd35	16292T 16497G 16519C	8584-10577-15326	R30
Kadmat	LKd43	16292T 16497G 16519C	8584-10577-15326	R30
Kadmat	LKd53	16292T 16497G 16519C	8584-10577-15326	R30
Kadmat	LKd54	16292T 16497G 16519C	8584-10577-15326	R30
Kadmat	LKd55	16292T 16497G 16519C	8584-10577-15326	R30
Kadmat	LKd57	16292T 16497G 16519C	8584-10577-15326	R30
Kadmat	LKd58	16292T 16497G 16519C	8584-10577-15326	R30
Kadmat	LKd59	16292T 16497G 16519C	8584-10577-15326	R30
Kadmat	LKd68	16292T 16497G 16519C	8584-10577-15326	R30
Kadmat	LKd69	16292T 16497G 16519C	8584-10577-15326	R30
Kadmat	LKd70	16292T 16497G 16519C	8584-10577-15326	R30
Kadmat	LKd71	16292T 16497G 16519C	8584-10577-15326	R30
Kadmat	LKd72	16292T 16497G 16519C	8584-10577-15326	R30
Kadmat	LKd73	16292T 16497G 16519C	8584-10577-15326	R30
Kadmat	LKd75	16292T 16497G 16519C	8584-10577-15326	R30
Kadmat	LKd79	16292T 16497G 16519C	8584-10577-15326	R30
Kadmat	LKd80	16292T 16497G 16519C	8584-10577-15326	R30
Kadmat	LKd81	16292T 16497G 16519C	8584-10577-15326	R30
Kadmat	LKd83	16292T 16497G 16519C	8584-10577-15326	R30
Kadmat	LKd87	16292T 16497G 16519C	8584-10577-15326	R30
Kadmat	LKd38	16292T 16497G 16519C 16519C	8584-10577-15326	R30
Kadmat	LKd41	16292T 16497G 16519C 16519C	8584-10577-15326	R30
Kadmat	LKd45	16292T 16497G 16519C 16519C	8584-10577-15326	R30
Kadmat	LKd46	16292T 16497G 16519C 16519C	8584-10577-15326	R30
Kadmat	LKd48	16292T 16497G 16519C 16519C	8584-10577-15326	R30
Kadmat	LKd49	16292T 16497G 16519C 16519C	8584-10577-15326	R30
Kadmat	LKd50	16292T 16497G 16519C 16519C	8584-10577-15326	R30
Kadmat	LKd39	16292T 16519C	8584-10577-15326	R30
Kadmat	LKd44	16172C 16274A 16519C	15326-15884	R31
Kadmat	LKd14	16172C 16274A 16519C	15326-15884	R31
Kadmat	LKd33	16192T 16311C 16497G 16519C	12308-12372-14364-15326	U1a1
Kadmat	LKd34	16497G 16519C	12308-12372-14364-15326	U1a1

Supplementary File

Kalpeni	LKL25	16223T 16356C	10398-10400-15043-15301	M
Kalpeni	LKL26	16223T 16356C	10398-10400-15043-15301	M
Kalpeni	LKL45	16223T 16356C	10398-10400-15043-15301	M
Kalpeni	LKL114	16187T 16223T 16274A 16289G	447G-10398-10400-13254-15043-15301-15670	M2b2
Kalpeni	LKL123	16187T 16223T 16274A 16289G 16319A 16320T	447G-10398-10400-13254-15043-15301-15670	M2b2
Kalpeni	LKL124	16187T 16223T 16274A 16289G 16319A 16320T	447G-10398-10400-13254-15043-15301-15670	M2b2
Kalpeni	LKL132	16187T 16223T 16274A 16289G 16319A 16320T	447G-10398-10400-13254-15043-15301-15670	M2b2
Kalpeni	LKL122	16187T 16223T 16274A 16289G 16319A 16320T 16362C	447G-10398-10400-13254-15043-15301-15670	M2b2
Kalpeni	LKL108	16187T 16223T 16274A 16289G 16319A 16320T 16362C	447G-10398-10400-13254-15043-15301-15670	M2b2
Kalpeni	LKL86	16187T 16274A 16289G 16319A 16320T 16362C	447G-10398-10400-13254-15043-15301-15670	M2b2
Kalpeni	LKL113	16187T 16223T 16274A 16289G 16362C	447G-10398-10400-13254-15043-15301-15670	M2b2
Kalpeni	LKL84	16187T 16223T 16274A 16289G 16319A 16320T 16362C	447G-10398-10400-13254-15043-15301-15670	M2b2
Kalpeni	LKL103	16126C 16223T	482-10398-10400-15043-15301	M3
Kalpeni	LKL106	16126C 16223T	482-10398-10400-15043-15301	M3
Kalpeni	LKL12	16126C 16223T	482-10398-10400-15043-15301	M3
Kalpeni	LKL120	16126C 16223T	482-10398-10400-15043-15301	M3
Kalpeni	LKL23	16126C 16223T	482-10398-10400-15043-15301	M3
Kalpeni	LKL36	16126C 16223T	482-10398-10400-15043-15301	M3
Kalpeni	LKL39	16126C 16223T	482-10398-10400-15043-15301	M3
Kalpeni	LKL43	16126C 16223T	482-10398-10400-15043-15301	M3
Kalpeni	LKL29	16126C 16223T 16287T	482-10398-10400-15043-15301	M3
Kalpeni	LKL37	16126C 16223T 16287T	482-10398-10400-15043-15301	M3
Kalpeni	LKL54	16126C 16223T 16287T	482-10398-10400-15043-15301	M3
Kalpeni	LKL118	16223T 16294T 16356C	195A-10398-10400-15043-15301-15431	M30
Kalpeni	LKL111	16223T 16234T	195A-10398-10400-15043-15301-15431	M30
Kalpeni	LKL129	16223T 16234T	195A-10398-10400-15043-15301-15431	M30
Kalpeni	LKL131	16223T 16234T	195A-10398-10400-15043-15301-15431	M30
Kalpeni	LKL128	16223T 16234T 16294T	195A-10398-10400-15043-15301-15431	M30
Kalpeni	LKL91	16223T 16234T 16294T 16356C	195A-10398-10400-15043-15301-15431	M30
Kalpeni	LKL115	16223T 16234T 16292T	195A-10398-10400-15043-15301-15431	M30
Kalpeni	LKL109	16223T 16234T 16294T	195A-10398-10400-15043-15301-15431	M30
Kalpeni	LKL117	16223T 16234T 16294T	195A-10398-10400-15043-15301-15431	M30
Kalpeni	LKL102	16223T 16311C 16356C	195A-10398-10400-15043-15301-15431	M30
Kalpeni	LKL110	16223T 16311C 16356C	195A-10398-10400-15043-15301-15431	M30
Kalpeni	LKL32	16223T 16311C 16356C	195A-10398-10400-15043-15301-15431	M30
Kalpeni	LKL40	16223T 16311C 16356C	195A-10398-10400-15043-15301-15431	M30
Kalpeni	LKL41	16223T 16311C 16356C	195A-10398-10400-15043-15301-15431	M30
Kalpeni	LKL21	16223T 16311C 16356C	195A-10398-10400-15043-15301-15431	M30
Kalpeni	LKL78	16048A 16129A 16223T 16390A	1888-10398-10400-15043-15301	M5
Kalpeni	LKL94	16223T	10398-10400-15043-15301-15968	M64
Kalpeni	LKL17	16223T	10398-10400-15043-15301-15968	M64
Kalpeni	LKL125	16153A	15326	R
Kalpeni	LKL127	rCRS	15326	R
Kalpeni	LKL130	16177G 16292T 16497C	8584-10577-15326	R30
Kalpeni	LKL119	16292T	8584-10577-15326	R30
Kalpeni	LKL116	16292T 16497C	8584-10577-15326	R30

Supplementary File

Kalpeni	LKL107	16292T 16497C	8584-10577-15326	R30
Kalpeni	LKL58	16292T 16497C	8584-10577-15326	R30
Kalpeni	LKL59	16292T 16497C	8584-10577-15326	R30
Kalpeni	LKL89	16292T 16497C	8584-10577-15326	R30
Kalpeni	LKL105	16292T 16497G	8584-10577-15326	R30
Kalpeni	LKL52	16292T 16497G	8584-10577-15326	R30
Kalpeni	LKL104	16292T 16497G	8584-10577-15326	R30
Kalpeni	LKL112	16292T 16497G	8584-10577-15326	R30
Kalpeni	LKL57	16292T 16497G	8584-10577-15326	R30
Kalpeni	LKL133	16292T 16352C 16497C	8584-10577-15326	R30
Kalpeni	LKL95	16111T 16356C 16362C	499-1811-12308-12372-15326-15693	U4
Kalpeni	LKL38	16111T 16181C 16246G 16258C 16356C 16362C	499-1811-12308-12372-15326-15693	U4
Kalpeni	LKL15	16111T 16339A 16356C 16362C	499-1811-12308-12372-15326-15693	U4
Kalpeni	LKL30	16111T 16124G 16241G 16276C 16301T 16356C 16362C	499-1811-12308-12372-15326-15693	U4
Kalpeni	LKL62	16111T 16124G 16241G 16276C 16356C 16362C	499-1811-12308-12372-15326-15693	U4
Kalpeni	LKL13	16111T 16124G 16241G 16276C 16301T 16356C 16362C	499-1811-12308-12372-15326-15693	U4
Kalpeni	LKL63	16111T 16311C 16356C 16362C	499-1811-12308-12372-15326-15693	U4
Kalpeni	LKL20	16111T 16356C 16362C	499-1811-12308-12372-15326-15693	U4
Kalpeni	LKL28	16111T 16356C 16362C	499-1811-12308-12372-15326-15693	U4
Kalpeni	LKL100	16111T 16356C 16362C	499-1811-12308-12372-15326-15693	U4
Kalpeni	LKL14	16111T 16356C 16362C	499-1811-12308-12372-15326-15693	U4
Kalpeni	LKL16	16111T 16356C 16362C	499-1811-12308-12372-15326-15693	U4
Kalpeni	LKL22	16111T 16356C 16362C	499-1811-12308-12372-15326-15693	U4
Kalpeni	LKL27	16111T 16356C 16362C	499-1811-12308-12372-15326-15693	U4
Kalpeni	LKL34	16111T 16356C 16362C	499-1811-12308-12372-15326-15693	U4
Kalpeni	LKL35	16111T 16356C 16362C	499-1811-12308-12372-15326-15693	U4
Kalpeni	LKL44	16111T 16356C 16362C	499-1811-12308-12372-15326-15693	U4
Kalpeni	LKL46	16111T 16356C 16362C	499-1811-12308-12372-15326-15693	U4
Kalpeni	LKL56	16111T 16356C 16362C	499-1811-12308-12372-15326-15693	U4
Kalpeni	LKL60	16111T 16356C 16362C	499-1811-12308-12372-15326-15693	U4
Kalpeni	LKL61	16111T 16356C 16362C	499-1811-12308-12372-15326-15693	U4
Kalpeni	LKL64	16111T 16356C 16362C	499-1811-12308-12372-15326-15693	U4
Kalpeni	LKL79	16111T 16356C 16362C	499-1811-12308-12372-15326-15693	U4
Kalpeni	LKL80	16111T 16356C 16362C	499-1811-12308-12372-15326-15693	U4
Kalpeni	LKL81	16111T 16356C 16362C	499-1811-12308-12372-15326-15693	U4
Kalpeni	LKL82	16111T 16356C 16362C	499-1811-12308-12372-15326-15693	U4
Kalpeni	LKL85	16111T 16356C 16362C	499-1811-12308-12372-15326-15693	U4
Kalpeni	LKL87	16111T 16356C 16362C	499-1811-12308-12372-15326-15693	U4
Kalpeni	LKL88	16111T 16356C 16362C	499-1811-12308-12372-15326-15693	U4
Kalpeni	LKL90	16111T 16356C 16362C	499-1811-12308-12372-15326-15693	U4
Kalpeni	LKL92	16111T 16356C 16362C	499-1811-12308-12372-15326-15693	U4
Kalpeni	LKL99	16111T 16356C 16362C	499-1811-12308-12372-15326-15693	U4
Kalpeni	LKL24	16111T 16356C 16362C	499-1811-12308-12372-15326-15693	U4
Kalpeni	LKL33	16111T 16356C 16362C	499-1811-12308-12372-15326-15693	U4
Kalpeni	LKL83	16111T 16356C 16362C	499-1811-12308-12372-15326-15693	U4
Kalpeni	LKL126	16111T 16356C 16362C	499-1811-12308-12372-15326-15693	U4
Kalpeni	LKL77	16111T 16356C 16362C	499-1811-12308-12372-15326-15693	U4
Kalpeni	LKL121	16153A 16318T	1811-12308-12372-14569-15326	U7

Kiltan	LKi18	16223T 16519C	10398-10400-15043-15301	M
Kiltan	LKi53	16187T 16223T 16274A 16289G 16319A 16320T 16362C	447G-10398-10400-13254-15043-15301-15670	M2b2
Kiltan	LKi54	16187T 16223T 16274A 16289G 16319A 16320T 16362C	447G-10398-10400-13254-15043-15301-15670	M2b2
Kiltan	LKi56	16187T 16223T 16274A 16289G 16319A 16320T 16362C	447G-10398-10400-13254-15043-15301-15670	M2b2
Kiltan	LKi57	16187T 16223T 16274A 16289G 16319A 16320T 16362C	447G-10398-10400-13254-15043-15301-15670	M2b2
Kiltan	LKi58	16187T 16223T 16274A 16289G 16319A 16320T 16362C	447G-10398-10400-13254-15043-15301-15670	M2b2
Kiltan	LKi61	16187T 16223T 16274A 16289G 16319A 16320T 16362C	447G-10398-10400-13254-15043-15301-15670	M2b2
Kiltan	LKi63	16187T 16223T 16274A 16289G 16319A 16320T 16362C	447G-10398-10400-13254-15043-15301-15670	M2b2
Kiltan	LKi65	16187T 16223T 16274A 16289G 16319A 16320T 16362C	447G-10398-10400-13254-15043-15301-15670	M2b2
Kiltan	LKi66	16187T 16223T 16274A 16289G 16319A 16320T 16362C	447G-10398-10400-13254-15043-15301-15670	M2b2
Kiltan	LKi67	16187T 16223T 16274A 16289G 16319A 16320T 16362C	447G-10398-10400-13254-15043-15301-15670	M2b2
Kiltan	LKi68	16187T 16223T 16274A 16289G 16319A 16320T 16362C	447G-10398-10400-13254-15043-15301-15670	M2b2
Kiltan	LKi52	16187T 16223T 16274A 16289G 16319A 16320T 16362C	447G-10398-10400-13254-15043-15301-15670	M2b2
Kiltan	LKi1	16187T 16223T 16274A 16289G 16319A 16320T 16362C	447G-10398-10400-13254-15043-15301-15670	M2b2
Kiltan	LKi6	16187T 16223T 16274A 16289G 16319A 16320T 16362C	447G-10398-10400-13254-15043-15301-15670	M2b2
Kiltan	LKi21	1618716223T 16274A 16289G 16319A 16320T 16362C	447G-10398-10400-13254-15043-15301-15670	M2b2
Kiltan	LKi30	16187T 1622316274A 16289G 16319A 16320T 16362C	447G-10398-10400-13254-15043-15301-15670	M2b2
Kiltan	LKi31	16187T 1622316274A 16289G 16319A 16320T 16362C	447G-10398-10400-13254-15043-15301-15670	M2b2
Kiltan	LKi70	16187T 16223T 16274A 16289G 16319A 16320T 16362C	447G-10398-10400-13254-15043-15301-15670	M2b2
Kiltan	LKi73	16187T 16223T 16274A 16289G 16319A 16320T 16362C	447G-10398-10400-13254-15043-15301-15670	M2b2
Kiltan	LKi26	16187T 16223T 16274A 16289G 16319A 16320T 16362C	447G-10398-10400-13254-15043-15301-15670	M2b2
Kiltan	LKi23	16187T 16223T 16274A 16289G 16319A 16320T 16362C	447G-10398-10400-13254-15043-15301-15670	M2b2
Kiltan	LKi29	16187T 16223T 16274A 16289G 16319A 16320T 16362C	447G-10398-10400-13254-15043-15301-15670	M2b2
Kiltan	LKi74	16187T 16223T 16274A 16289G 16319A 16320T 16362C	447G-10398-10400-13254-15043-15301-15670	M2b2
Kiltan	LKi79	16187T 16223T 16274A 16289G 16319A 16320T 16362C	447G-10398-10400-13254-15043-15301-15670	M2b2
Kiltan	LKi47	16187T 16223T 16274A 16289G 16319A 16320T 16362C	447G-10398-10400-13254-15043-15301-15670	M2b2
Kiltan	LKi81	16187T 16223T 16274A 16289G 16319A 16320T 16362C	447G-10398-10400-13254-15043-15301-15670	M2b2
Kiltan	LKi84	16187T 16223T 16274A 16289G 16319A 16320T 16362C	447G-10398-10400-13254-15043-15301-15670	M2b2
Kiltan	LKi41	16223T 16274A 16289G 16319A 16320T 16362C 16519C	447G-10398-10400-13254-15043-15301-15670	M2b2
Kiltan	LKi45	16223T 16234T 16289G 16294T 16519C	195A-10398-10400-15043-15301-15431	M30
Kiltan	LKi33	16223T 16234T 16294T 16519C	195A-10398-10400-15043-15301-15431	M30
Kiltan	LKi38	16223T 16519C	482-10398-10400-15043-15301	M3c
Kiltan	LKi69	16223T 16231C 16362C	461-10398-10400-15043-15301	M6a
Kiltan	LKi80	16223T 16231C 16311C 16362C	461-10398-10400-15043-15301	M6a
Kiltan	LKi32	16223T 16231C 16311C 16362C 16519C	461-10398-10400-15043-15301	M6a
Kiltan	LKi43	16223T 16231C 16311C 16362C 16519C	461-10398-10400-15043-15301	M6a
Kiltan	LKi82	16223T 16231C 16294T 16519C	461-10398-10400-15043-15301	M6a
Kiltan	LKi35	16355T 16519C	15326	R
Kiltan	LKi15	16292T 16497G 16519C	8584-10577-15326	R30
Kiltan	LKi4	16223T 16292T 16497G 16519C	8584-10577-15326	R30
Kiltan	LKi8	16292T	8584-10577-15326	R30
Kiltan	LKi9	16292T 16497	8584-10577-15326	R30
Kiltan	LKi49	16292T 16497G 16519C	8584-10577-15326	R30

Supplementary File

Kiltan	LKi50	16292T 16497G 16519C	8584-10577-15326	R30
Kiltan	LKi51	16292T 16497G 16519C	8584-10577-15326	R30
Kiltan	LKi55	16292T 16497G 16519C	8584-10577-15326	R30
Kiltan	LKi59	16292T 16497G 16519C	8584-10577-15326	R30
Kiltan	LKi60	16292T 16497G 16519C	8584-10577-15326	R30
Kiltan	LKi64	16292T 16497G 16519C	8584-10577-15326	R30
Kiltan	LKi17	16292T 16497G 16519C	8584-10577-15326	R30
Kiltan	LKi24	16292T 16497G 16519C	8584-10577-15326	R30
Kiltan	LKi27	16292T 16497G 16519C	8584-10577-15326	R30
Kiltan	LKi20	16093C 16292T 16497G 16519C	8584-10577-15326	R30
Kiltan	LKi25	16292T 16497G	8584-10577-15326	R30
Kiltan	LKi71	16292T 16497G 16519C	8584-10577-15326	R30
Kiltan	LKi72	16292T 16497G 16519C	8584-10577-15326	R30
Kiltan	LKi75	16292T 16497G 16519C	8584-10577-15326	R30
Kiltan	LKi76	16292T 16497G 16519C	8584-10577-15326	R30
Kiltan	LKi77	16292T 16497G 16519C	8584-10577-15326	R30
Kiltan	LKi40	16292T 16362C 16399T 16519C	8584-10577-15326	R30
Kiltan	LKi44	16292T 16497G 16519C	8584-10577-15326	R30
Kiltan	LKi37	16093C 16223T 16292T 16497G 16519C	8584-10577-15326	R30
Kiltan	LKi2	16172C 1618516223T 16261T 16304C 16362C 16519C	15326-15884	R31
Kiltan	LKi78	16172C 16185T 16261T 16204C 16362C 16519C	15326-15884	R31
Kiltan	LKi28	16172C 16185T 16261T 16304C 16362C 16519C	15326-15884	R31
Kiltan	LKi39	16172C 16185T 16187T 16261T 16304C 16362C 16519C	15326-15884	R31
Kiltan	LKi5	16192T 16304C 16311C	14544-15326	R5
Kiltan	LKi85	16192T 16304C 16311C	14544-15326	R5
Kiltan	LKi86	16192T 16304C 16311C	14544-15326	R5
Kiltan	LKi88	16519C	9449-15326	R8b
Minicoy	M26	16111T 16192T 16223T 16293G	10398-10400-15043-15301	M
Minicoy	M102	16223T	10398-10400-15043-15301	M
Minicoy	M110	16223T 16293G	10398-10400-15043-15301	M
Minicoy	M42	16223T 16293G	10398-10400-15043-15301	M
Minicoy	M59	16129A 16223T 16234T	1888-10398-10400-15043-15301	M5a2a
Minicoy	M86	16223T	10398-10400-15043-15301-15968	M64
Minicoy	M88	16223T	10398-10400-15043-15301-15968	M64
Minicoy	M105	16223T	10398-10400-15043-15301-15968	M64
Minicoy	M118	16223T 16527T	10398-10400-15043-15301-15968	M64
Minicoy	M113	16184T 16215G 16215G 16223T 16311C	9061-10398-10400-15043-15301	M66
Minicoy	M114	16184T 16215G 16215G 16223T 16311C	9061-10398-10400-15043-15301	M66
Minicoy	M115	16184T 16215G 16223T	9061-10398-10400-15043-15301	M66
Minicoy	M77	16184T 16215G 16223T	9061-10398-10400-15043-15301	M66
Minicoy	M84	16184T 16215G 16223T	9061-10398-10400-15043-15301	M66
Minicoy	M116	16184T 16215G 16223T 16311C	9061-10398-10400-15043-15301	M66
Minicoy	M119	16184T 16215G 16223T 16311C	9061-10398-10400-15043-15301	M66
Minicoy	M51	16184T 16215G 16223T 16311C	9061-10398-10400-15043-15301	M66
Minicoy	M35	16184T 16215G 16223T 16311C	9061-10398-10400-15043-15301	M66
Minicoy	M78	16184T 16223T	9061-10398-10400-15043-15301	M66
Minicoy	M34	16184T 16223T 16311C	9061-10398-10400-15043-15301	M66
Minicoy	M66	16051G 16223T 16231C 16311C 16362C	461-10398-10400-15043-15301	M6a

Supplementary File

Minicoy	M111	16223T 16294T 16294T 16295T	10398-10400-12091-15043-15301	M7c
Minicoy	M95	16051G 16223T 16294T 16295T	10398-10400-12091-15043-15301	M7c
Minicoy	M96	16223T 16294T 16295T	10398-10400-12091-15043-15301	M7c
Minicoy	M101	16223T 16295T	10398-10400-12091-15043-15301	M7c
Minicoy	M109	16223T 16295T	10398-10400-12091-15043-15301	M7c
Minicoy	M94	16223T 16295T	10398-10400-12091-15043-15301	M7c
Minicoy	M103	16223T 16295T 16295T	10398-10400-12091-15043-15301	M7c
Minicoy	M106	16223T 16295T 16295T	10398-10400-12091-15043-15301	M7c
Minicoy	M107	16223T 16295T 16295T	10398-10400-12091-15043-15301	M7c
Minicoy	M39	16187T 16241T 16319A 16342C	15326	R
Minicoy	M108	16129H 16301T	15326	R
Minicoy	M80	rCRS	15326	R
Minicoy	M98	rCRS	15326	R
Minicoy	M99	rCRS	15326	R
Minicoy	M74	16189C	15326	R
Minicoy	M90	16189C	15326	R
Minicoy	M108	16233T 16292T	8584-10577-15326	R30
Minicoy	M62	16129A 16172C 16301T	15326-15884	R31
Minicoy	M41	16192T 16304C 16311C 16356C 16524G	14544-15326	R5
Minicoy	M73	16051G	1811-12308-12372-15326	U2
Minicoy	M92	16247G 16254G	1811-12308-12372-15326	U2c1
Minicoy	M29	16051G 16247G 16254G	1811-12308-12372-15326	U2c1
Minicoy	M55	16051G 16247G 16254G	1811-12308-12372-15326	U2c1
Minicoy	M61	16051G 16247G 16254G	1811-12308-12372-15326	U2c1
Minicoy	M64	16051G 16247G 16254G	1811-12308-12372-15326	U2c1
Minicoy	M85	16051G 16247G 16254G	1811-12308-12372-15326	U2c1
Minicoy	M87	16051G 16247G 16254G	1811-12308-12372-15326	U2c1
Minicoy	M93	16051G 16247G 16254G	1811-12308-12372-15326	U2c1
Minicoy	M67	16051G 16247G 16254G 16362C	1811-12308-12372-15326	U2c1
Minicoy	M31	16051G 16234T 16247G 16254G	1811-12308-12372-15326	U2c1
Minicoy	M32	16051G 16234T 16247G 16254G	1811-12308-12372-15326	U2c1
Minicoy	M37	16051G 16234T 16247G 16254G	1811-12308-12372-15326	U2c1
Minicoy	M27	16111T 16356C 16362C	499-1811-12308-12372-15326-15693	U4
Minicoy	M33	16111T 16356C 16362C	499-1811-12308-12372-15326-15693	U4
Minicoy	M71	16051G 16176T 16193T 16278T 16357C	499-12308-12372-15326	U9a1
Minicoy	M43	16051G 16193T 16215G 16357C	499-12308-12372-15326	U9a1
Minicoy	M56	16051G 16193T 16192T 16278T 16357C	499-12308-12372-15326	U9a1
Minicoy	M104	16051G 16193T 16278T 16357C	499-12308-12372-15326	U9a1
Minicoy	M45	16051G 16193T 16278T 16357C	499-12308-12372-15326	U9a1
Minicoy	M48	16051G 16193T 16278T 16357C	499-12308-12372-15326	U9a1
Minicoy	M49	16051G 16193T 16278T 16357C	499-12308-12372-15326	U9a1
Minicoy	M50	16051G 16193T 16278T 16357C	499-12308-12372-15326	U9a1
Minicoy	M53	16051G 16193T 16278T 16357C	499-12308-12372-15326	U9a1
Minicoy	M54	16051G 16193T 16278T 16357C	499-12308-12372-15326	U9a1
Minicoy	M58	16051G 16193T 16278T 16357C	499-12308-12372-15326	U9a1
Minicoy	M60	16051G 16193T 16278T 16357C	499-12308-12372-15326	U9a1
Minicoy	M65	16051G 16193T 16278T 16357C	499-12308-12372-15326	U9a1
Minicoy	M68	16051G 16193T 16278T 16357C	499-12308-12372-15326	U9a1

Supplementary File

Minicoy	M69	16051G 16193T 16278T 16357C	499-12308-12372-15326	U9a1
Minicoy	M75	16051G 16193T 16278T 16357C	499-12308-12372-15326	U9a1
Minicoy	M81	16051G 16193T 16278T 16357C	499-12308-12372-15326	U9a1
Minicoy	M82	16051G 16193T 16278T 16357C	499-12308-12372-15326	U9a1

Supplementary Table 2. The mtDNA (left) and Y chromosome (right) haplogroups frequency in Lakshadweep and surrounding regions

	Gujarat	Andhra Pradesh	Kerala	Sri Lanka	Maldives	Lakshadweep
n	125	524	301	231	130	555
**C4**	-	-	0.02	-	-	-
**H**	0.02	-	-	-	-	-
**HV**	-	0.01	-	0.03	0.01	-
**J1**	0.02	-	-	-	-	-
**J2**	-	0.01	-	-	-	-
**K**	-	0.01	-	-	-	-
**M**	0.16	0.20	0.02	0.03	0.04	0.03
**M2**	0.01	0.10	0.32	-	-	0.19
**M3**	0.14	0.10	0.09	0.03	-	0.02
**M4**	0.02	0.04	0.05	-	-	0.01
**M5**	0.07	0.06	0.09	0.04	-	0.01
**M6**	0.01	0.06	-	0.08	0.08	0.02
**M7**	-	-	-	-	-	0.02
**M18**	0.01	0.04	-	0.03	-	-
**M25**	-	0.02	-	-	-	-
**M30**	0.02	0.01	0.25	0.03	0.09	0.08
**M33**	0.01	0.01	0.03	0.06	0.04	0.00
**M35**	-	0.02	0.02	0.03	0.02	0.00
**M36**	-	-	0.02	0.02	0.08	-
**M38**	-	0.01	-	0.01	-	-
**M39**	0.01	0.02	-	-	0.07	-
**M40**	0.01	-	0.01	-	-	-
**M41**	-	-	-	0.01	0.05	-
**M44**	-	0.01	-	-	-	-
**M45**	-	-	-	0.01	-	-
**M4'67**	-	-	-	-	0.01	0.02

	Gujarat	Andhra Pradesh	Kerala	Sri Lanka	Maldives	Lakshadweep
n	447	635	121	74	124	166
C(M216)	0.08	0.04	0.12	-	0.01	-
F(M89)	0.03	0.04	0.26	0.09	0.06	-
G(M201)	-	-	-	-	0.01	-
H(M69)	0.33	0.02	0.08	0.01	0.06	-
H1(M52)	-	0.13	0.25	-	0.08	-
H1a(M82)	-	0.09	-	0.14	-	0.06
H2(Apt)	-	0.01	0.04	-	-	-
J(M304)	-	-	0.01	-	-	0.01
J2a(M172)	0.11	0.08	0.02	0.14	0.21	0.11
K(M9)	0.02	0.05	-	0.01	0.04	-
T(M70)	0.00	0.03	-	-	-	-
L(M11)	0.09	0.09	0.07	0.19	0.14	-
O2a(M95)	-	0.06	-	-	-	-
O3(M122)	-	0.01	-	-	-	-
P(M45)	0.02	0.01	-	0.03	-	-
Q(M242)	0.02	0.00	-	-	0.03	-
R(M207)	0.00	0.01	-	-	-	-
R1(M173)	0.01	0.01	-	-	-	0.08
R1a1(M17)	0.21	0.16	0.07	0.27	0.24	0.31
R1b(M269)	-	0.03	-	-	-	-
R2a(M124)	0.08	0.12	0.07	0.12	0.12	0.42

Supplementary File

**M66**	-	-	-	0.02	0.08	0.02
**M52**	0.01	-	-	-	0.01	-
**N**	0.02	-	-	0.01	0.01	-
**N5**	-	-	-	0.01	-	-
**R**	0.14	0.07	0.02	-	0.08	0.12
**R2**	0.03	-	-	-	-	-
**R5**	0.02	0.07	0.01	0.06	0.05	0.01
**R6**	-	0.01	0.01	0.02	-	-
**R7**	-	0.01	-	0.02	-	-
**R8**	-	-	-	0.01	-	0.00
**R30**	-	-	0.03	0.18	-	0.31
**R31**	-	-	-	-	0.01	0.01
**T**	-	0.01	-	0.01	-	-
**U**	0.02	-	-	-	-	-
**U1**	-	-	0.02	0.06	0.07	0.01
**U2**	0.03	0.03	-	0.04	-	0.02
**U2a**	0.02	0.04	-	-	0.06	-
**U2b**	0.02	-	-	-	-	-
**U2c**	0.01	0.01	-	-	0.12	-
**U4**	-	-	0.01	-	0.02	0.08
**U5**	0.01	0.01	-	0.01	-	-
**U7**	0.14	0.02	-	0.10	-	-
**U9**	-	0.01	-	-	0.02	0.03
**W**	0.05	0.01	-	0.01	-	-

Supplementary Table 3. The detailed Y chromosome genotyping results of various Lakshadweep Islands

**Island name**	***n***	**H1a-M82**	**J-M304**	**J2a-M172**	**R1a-M17**	**R2a-M124**
Amini	44	1	0	5	17	21
Kadmat	24	1	1	4	8	10
Kiltan	34	1	0	4	17	12
Chetlat	23	2	0	2	9	10
Bitra	7	0	0	2	1	4
Minicoy	12	3	0	0	3	6
Agatti	11	0	0	2	5	4
Kavaratti	4	1	0	0	1	2
Androth	7	0	0	0	2	5
